# Flexibility of Biodegradable Polymer Stents with Different Strut Geometries

**DOI:** 10.3390/ma13153332

**Published:** 2020-07-27

**Authors:** Chong Chen, Yan Xiong, Zhongyou Li, Yu Chen

**Affiliations:** 1 Department of Applied Mechanics, Sichuan University, Chengdu 610065, China; scuchenchong@outlook.com (C.C.); zhongyou_li@outlook.com (Z.L.); 2 School of Mechanical Engineering, Sichuan University, Chengdu 610065, China

**Keywords:** biodegradable stent, flexibility, experiment, numerical analysis

## Abstract

Objective: Biodegradable stents (BDSs) represent a new technological development in the field of cardiovascular angioplasty; good flexibility helps stents pass through tortuous vessels during delivery and reduces the amount of damage caused to blood vessels. This study investigates the relationship between flexibility and the geometry of BDS struts. Methods: Four stent struts with different geometry (circular, triangular, hexagonal, and spline curved) and the same links were modeled to evaluate their flexibility via a three-point bending experimental method and a numerical method. Results: The bending state of the four stents was well-balanced. The bending effect of the four stents was different. Under the same conditions, the circular and spline curved stents showed the best bending effects while the hexagonal stent was the worst. However, these differences were not significant. Conclusion: The flexibility of BDSs is related to the geometry of the struts and links; however, the geometry of the struts has less effect on flexibility than the links. The greater the area enclosed by the strut centerline, the better flexibility of the stent.

## 1. Introduction

Coronary stents are widely used to treat atherosclerotic vessel diseases. Their biomechanical properties are believed to have an important effect on acute and long-term clinical results after the implant procedure. Biodegradable stents (BDSs) have attracted attention over the last decade, following the recognition that stents are only needed temporarily in the body, since they can be fully absorbed. Poly-(L-lactic acid) (PLLA) has been widely used for different biomedical applications due to its mechanical behavior, biocompatibility, and biodegradability.

Stent properties include radial force and bulking, flexibility, and foreshortening. Flexibility is the ability to bend in order to accommodate an angle; this is important for stent delivery so they will conform to the vessel after implantation [1]. Good flexibility helps stents pass through tortuous blood vessels during delivery and reduces the stress between expanded stents and the surrounding tissue [2]. Previously, flexibility has been investigated through numerical simulations, but most of these studies have focused on drug-eluting stents (DESs) rather than of BDSs. Computational analyses provide a lot of information about stent structure under highly controlled conditions; this makes it possible to screen different and competing coronary stent designs [3]. Furthermore, stent design is a major factor which determines their reliability during implantation in a blocked artery and throughout long-term in vivo service. Therefore, numerous studies have explored stent design. Petrini et al. [4] evaluated the index of flexibility for different stents when the material was elastically or plastically deformed. Ormiston et al. [5] compared the longitudinal flexibility of 13 stent designs before and after balloon expansion; they found that stiffness was least for the coil and hybrid designs, the MultiLink design was the most flexible, and the crown was the stiffest of the slotted tube designs. Azaouzi et al. [6] compared symmetric and asymmetric stent link designs for their flexibility. Ju et al. [7] found that link geometry had a significant effect on the bending behavior of stents. Syaifudin et al. [8] found that asymmetric stents are less flexible that sinusoidal stents in their unexpanded configurations. Bobel et al. [9] selected three physical stent geometries for comparative study and established the relationship between radial strength, flexibility, and longitudinal resistance. They found that it was possible to produce an ideal PLLA stent with mechanical performance comparable to existing designs. Gergory et al. [10] demonstrated the feasibility of biodegradable polylactic acid (PLA) biliary stents using a model of a porcine bile duct with a stricture. At the same time, some researchers have conducted experimental tests on the flexibility of different commercial stents. For example, Rieu et al. [11] carried out a comparative analysis of coronary stent assessments and evaluations based on the trackability, flexibility, and conformability. Mori et al. [12] employed a four-point bending test and the finite element method to evaluate stent flexibility. Li et al. [13] found that optimization theory is a very useful and efficient way to study coronary artery stents. Shah et al. [14] reported a case where longitudinal deformation of a coronary stent was observed while treating an ostial lesion and aneurysm of the left anterior descending artery. Liu et al. [15] also investigated the relationship between the bending load and the structural parameters.

These contributions provide useful and valuable information about the structural design of cardiovascular implants; however, until now, little attention has been given to the flexibility of BDSs with different strut geometries. Because the radial strength of polymer stents is generally low, most biodegradable stents adopt a thicker structural design to improve the radial support force. The four stents we designed used a universal biodegradable stent thickness of 0.15 mm as in this study [16]. The results of our pervious study show that the radial performance of the four stents is satisfactory [17]. However, the increase of thickness will lead to the deterioration of the stent flexibility [9]. Based on this, this paper focuses on the flexibility of biodegradable stents. In this study, we used experimental and numerical methods to explore the structural behavior of stents and the effect of stent design. Stent struts with four different geometries were included and the BDS performance was assessed in terms of longitudinal flexibility, which affects the delivery and placement of intracoronary artery stents.

## 2. Methods

### 2.1. Models

This paper focuses on the effect of stent strut configuration on flexibility. The design of the four configurations involved in this paper takes many factors into consideration. First of all, we searched literatures on stents and summarized stent configurations. Since most of the configuration designs in the literature are metal stents, there is little information about the configuration of polymer biodegradable stents. The metal stents do not have the problem of radial strength while, at present, the biggest problem of polymer biodegradable stents is the low radial strength. In order to ensure the strong radial strength of biodegradable stents, our design increased the coverage as much as possible [6]. In the process of compression, the gap of the stents will gradually decrease and the structure will become compact. A stent should be compressed into the smallest diameter as possible (The smallest balloon diameter is 1.00 mm). This allows the stent to have a better trafficability in the process of implanting in blood vessels. When we crimp the stent from the diameter of 3.0 mm to 1.0 mm, in theory, coverage of the stent can only be designed up to 30%. If it is more than 30%, the stent will be squeezed between the structures during the compression process, which may do damage to the stent. In addition, in order to ensure that the stent can be crimped to a smaller diameter and not squeeze between the unit cells, the links should only be in a linear configuration. The reason is that the links will be as close as possible to the two struts of the stent after compression and occupying the minimum space. If links are designed with other curved configurations, the strut will squeeze links in advance during the compression process and damage the stent. The length of the links is expected to be the same as that of the stent strut after compression, so as to ensure the maximum coverage and not cause buckling of the stent. Combined with the radial strength problem, actual application environment and fabrication technology of fully degraded polymer stents, four stents in our study were finally optimized and screened out to compare their flexibility.

**Figure 1:**
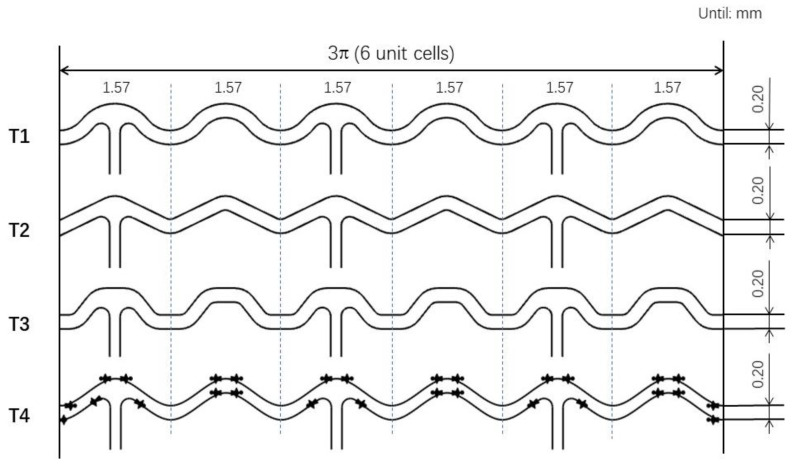
2D stent models: T1: circular strut, T2: triangular strut, T3: hexagonal strut, and T4: spline curve strut.

The stent considered in this study consists of 15 struts and 42 links. We refer to the parameters commonly used in biodegradable stents on the market [16]. Each strut consists of 6 valley-to-peak-to-valleys (stent unit cells) in one circumferential direction and is axially connected by the same straight lines. After expansion, the outer diameter of the stent is 3.0 mm. In addition, they are all 0.2 mm wide at the top and bottom of the cells. The differences between the four designs lie in the geometries of the stent struts. The stent unit cells in each of these four designs have the same length, height, and rounded corners. Figure 1 shows 2D models of stents with the different struts considered in this study: T1, T2, T3, and T4 are circular, triangular, hexagonal, and spline curve struts, respectively.

**Figure 2:**
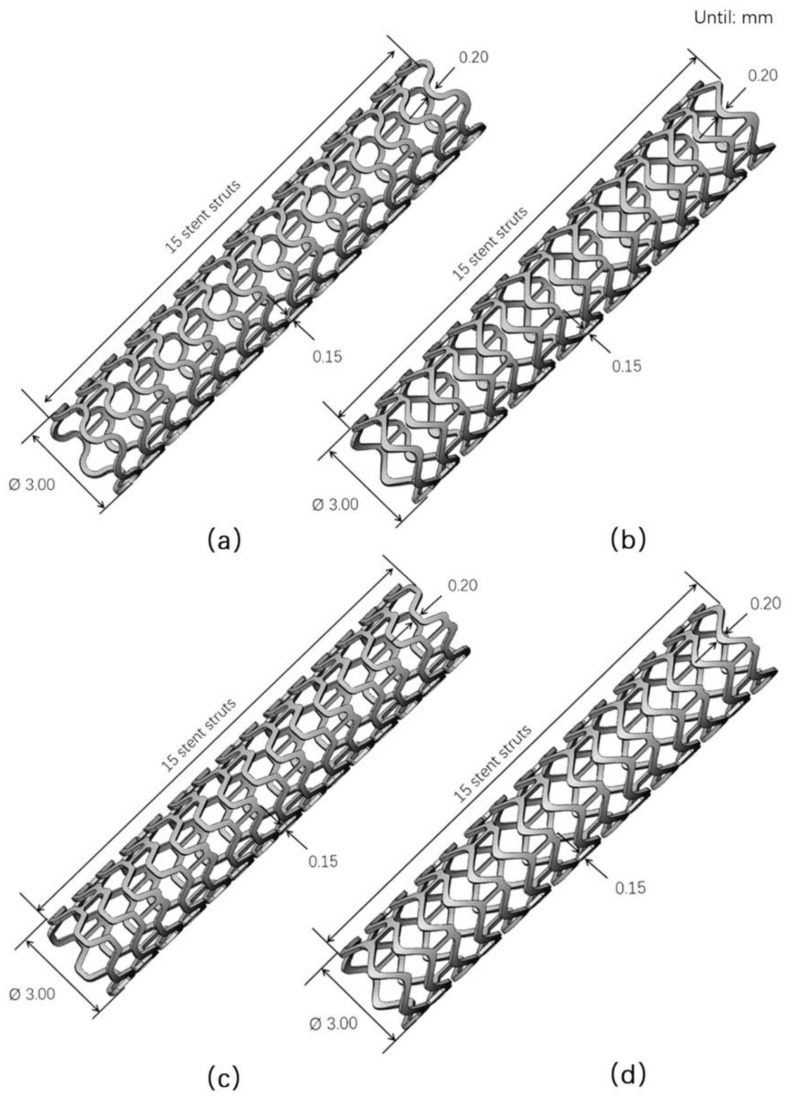
3D stent models: (**a**) T1: circular strut, (**b**) T2: triangular strut, (**c**) T3: hexagonal strut, and (**d**) T4: spline curve strut.

Four simplified 3D stent models were constructed using SolidWorks 2014 (Dassault Systèmes SolidWorks Corporation, Waltham, MA, USA). The stent models had a width of 0.2 mm and a thickness of 0.15 mm. The lengths of all the stents ranged from 17 to 18 mm, depending on the stent geometry. Figure 2 shows the 3D stent models.

### 2.2. Experimental Methods

The 2D sketches of the four BDSs were designed using AutoCAD (Autodesk, Inc, San Rafael, CA, USA), then converted to the DWG format. We imported the DWG files into CagilaV3 (CAM-Service GmbH Ges. für Software u. Automationstechnik, Hannover, Germany) software in order to obtain the NC files, then uploaded these to the laser system (s-pulse HP, Amplitude Systemes, Paris, France). The CAM system CAGILA is an innovative NC programming software for 2D beam cutting. The PLLA tubes were provided by the stent company Xingtai Pule^TM^ (Chengdu, Sichuan, China). The final stent models were produced by laser cutting. The stents were kept at a temperature of approximately 20 °C to avoid self-foreshortening.

**Figure 3:**
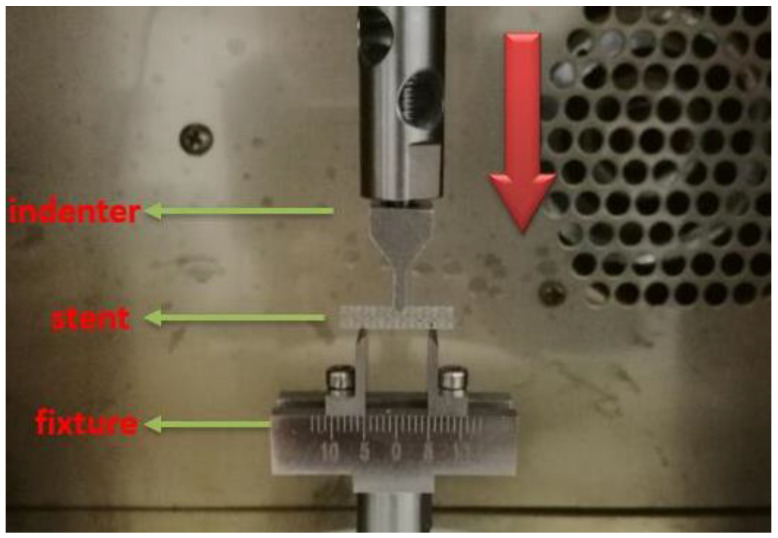
Three-point bending experiment.

The BOSE cupping machine (TA Instruments, New Castle, DE 19720, PA, USA) was used to examine the flexibility of the stents. The stents were fixed using a special fixture, as shown in Figure 3. Bending tests under displacement control were carried out. The bending flexibility of the BDSs was evaluated using the three-point method with a fixed span of 10 mm in the center of all the stents. The middle of each stent was pressed down by 2 mm using the indenter with the same waveform and frequency. Three groups of experiments were carried out for each kind of stent and in each group two sets of data were recorded at fifty time points. These data sets were the displacement and load of the contact point between the indenter and the stent during the bending process. We used this information to calculate the average values and to draw the curves.

### 2.3. Numerical Simulation

The computations were performed using the ABAQUS 6.13 (ABAQUS Inc., Providence, RI, USA) solver. To solve the problem of static equilibrium, the static procedure in the Abaqus/Standard finite element code was used (Newton–Raphson algorithm) [18]. In order to make the nonlinear analysis feasible and accurate, a large deformation analysis was performed and the final states of the stent were recorded.

**Figure 4:**
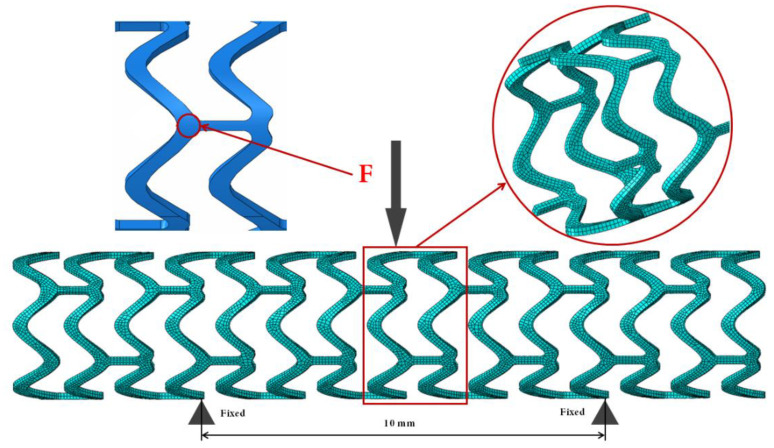
Three-point bending (including fixed constraint and force load) and mesh of the model.

To simplify the simulation process, we removed the fixture model and only the stent model was used. The locations that were in contact with the fixture during the experiment were fixed, as shown in Figure 4; the two points located 10 mm apart were at the bottom of the stents. A vertical downward force was applied at the middle of the top of the stent in order to bend it. The meshed model is shown in Figure 4.

According to the material information provided by the supplier: non-degraded PLLA was set to have a mass density of 1.28E-009 g/mm^3^, Young’s modulus *E* set to 3700 MPa, Poisson’s ratio *v* was 0.35, and the yield stress *δs* was 45 MPa. The Von Mises yield criterion and isotropic hardening criterion were used to describe the plastic deformation behavior of the stent.

For the large deformation analysis, a 0.2 N force was applied to the middle of the stents in order to obtain their final deformed states. The correct boundary conditions reduced excessive rigid body displacement and simplified the calculations, but they also reflected the true deformation of the stent as far as possible. The boundary constraints were imposed on the whole system in rectangular coordinates, where the 1, 2, and 3 directions represented the X, Y, and Z axial directions of the model system, respectively. The specific settings were as follows:

We constrained the Y-axis displacement of two fixed areas of the stent: U_2_ = 0. The magnitude of the concentrated force in the Y direction (CF_2_) that was applied to the middle of the top of the stent was determined by the bending process.

To investigate the effect of the flexibility on the four stents during bending, the stents were modeled by 3D 8-node brick “reduced integration” elements (C3D8R) using ABAQUS finite element code. The refinement of the meshes did not stop until the computational results reached grid-independence. Finite element meshes consisting of 142,756, 134,584, 170,564, and 140,001 elements were used for T1, T2, T3, and T4, respectively. The Von Mises stress after bending was analyzed for each stent.

## 3. Results

The stents need to adapt to the complex bending environment inside the vessels. Flexibility is considered to be an important characteristic of stent design. Good flexibility can ensure the stent goes through the complex vascular path successfully under the guidance of the catheter system, and can also make the stent fully fit with the blood vessels after expansion, reducing the mechanical damage to blood vessel walls [12]. The three point bending method can reflect the compliance of the support to a certain extent and the test results are discussed in the following paragraphs.

### 3.1. Bending Process Data

**Figure 5:**
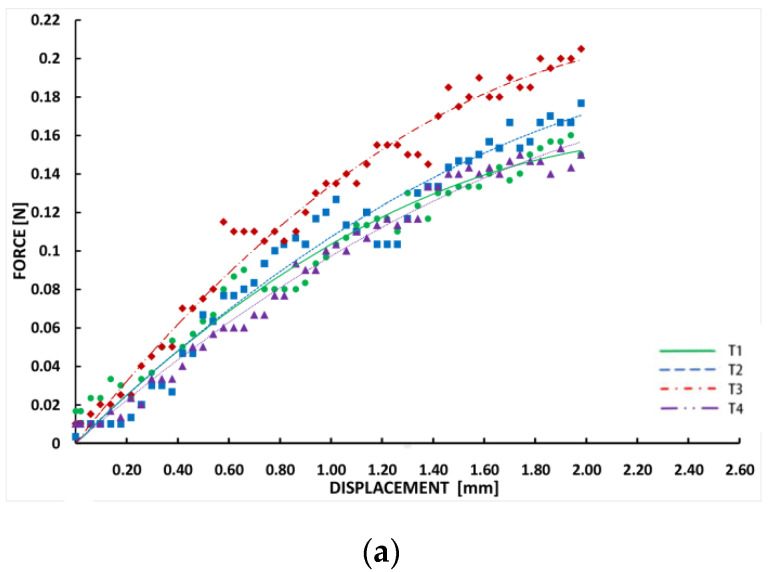

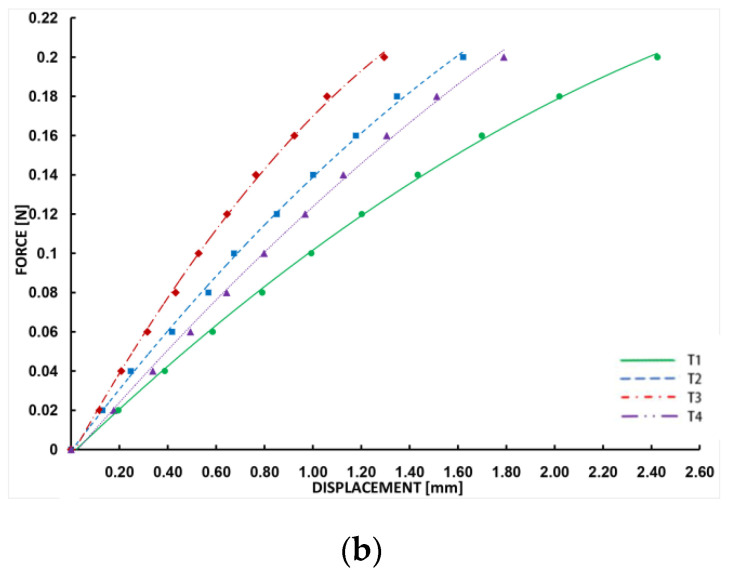
Displacement-force data from the 3.0 mm diameter biodegradable stents: (**a**) experiment results and (**b**) simulation results.

The experimental and simulation flexibility results for the four BDS models with different struts are shown in Figure 5. The relationship between the displacement and the force corresponding to the four different stent designs during the bending process is presented. The force in the simulation was defined as the downward pressure from the indenter on the stent in the experiment. Displacement represents the displacement of the loading position of the stent in the direction of the load.

The experimental results show that the curves for all of the stents show a consistent trend. As the force increases, the displacement and the displacement growth rate increase. The trend lines of T1 and T4 grow fastest, indicating that they are more flexible. T2 shows slightly less flexibility than T1 and T4, while T3 presents the poorest flexibility. The flexibility of the four stents was ranked as: T1≈T4>T2>T3. Despite this, the differences in the flexibility of the four stents was not significant. The simulation results are similar to the experimental results; they show a consistent trend with T1 increasing fastest and no substantial differences between the other stents. The flexibility of the four stents are ranked as: T1>T4>T2>T3. Likewise, these differences are not significant.

### 3.2. Stress Distribution

**Figure 6:**
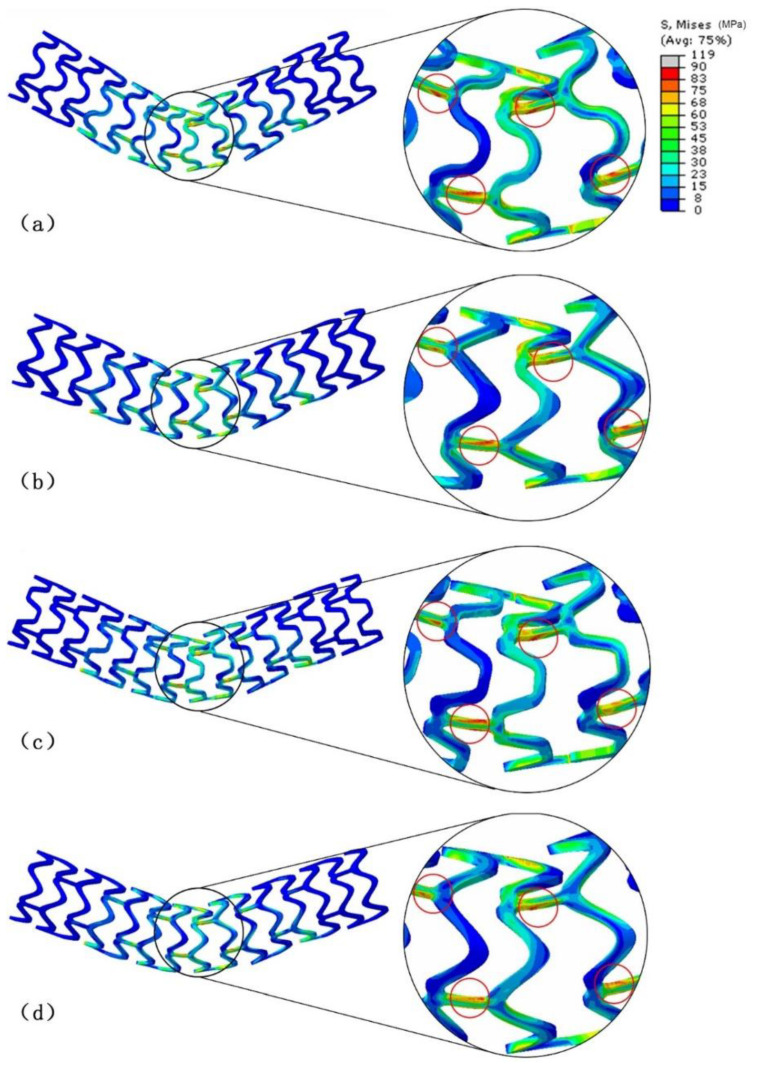
Stress distribution and deformation of the four models under the bending load: (**a**) T1: circular strut, (**b**) T2: triangular strut, (**c**) T3: hexagonal strut, and (**d**) T4: spline curved strut.

Figure 6 shows the stent deformation and Von-Mises stresses (MPa) obtained at the end of the simulation for each of the four stents.

The three-point bending method was used to study flexibility of the stents. The four stents were in a good bending state. Due to the boundary conditions, the maximum stress occurred in the two fixed areas at the bottom of the stents and in the area under direct force. Outside of these areas, the peak stress was mainly concentrated on the links with the largest deformation as indicated by the red circle in Figure 6. The deformation and stress of the stent struts was not very high.

**Figure 7:**
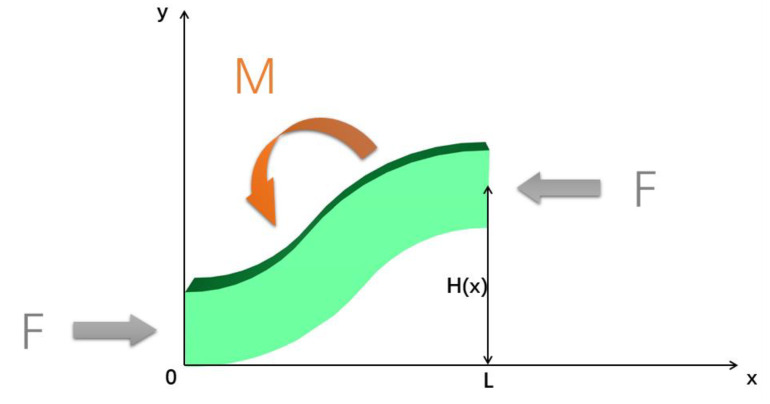
Stent during the bending process.

We also found that during the process of bending under force, when the area enclosed by the strut is larger, the flexibility of the stent is improved. This is due to the axisymmetric structure of the stent strut; we just used half of it for the analysis. The force analysis on T1 is shown in Figure 7. The force analysis for stents T2, T3 and T4 was similar to that for T1.

As shown in Figure 7, H(x) represents the distance from the midpoint of the stent to the X-axis. The constants *F* and *L* represent the external force and struts length, respectively. Furthermore, *M* is the bending moment; this is what caused the stent to bend. The bending moment for a stent is given by

(1) M=F×H(x)

The average moment of the support $\bar{M}$ is given by

(2) $$\bar{M}=\frac{\int_{0}^{X}F\times H\left(x\right)}{L}dx$$

and $\bar{M}$ is given by

(3) $$\bar{M}=F\times \frac{A}{L}$$

**Figure 8:**
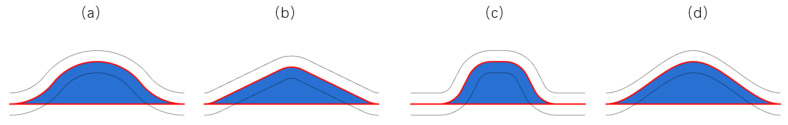
Area enclosed by the centerline of the four stent struts (blue areas enclosed by red lines): (**a**) T1: circular strut, (**b**) T2: triangular strut, (**c**) T3: hexagonal strut, and (**d**) T4: spline curved strut.

Here, *A* is defined as the area enclosed by the central axis of the stent strut and the X-axis. According this analysis, the flexibility of the bending stent is related to *A*. Hence, we extracted the areas enclosed by the center line and the middle line of struts, as shown in Figure 8.

The occupied areas were calculated by integrated computation. The areas in T1, T2, T3, and T4 were 0.29924384, 0.260726949, 0.22443287, and 0.299354584 mm^2^, respectively. The blue area shows the relationship between them, which can be expressed as

(4) T1≈T4>T2>T3

## 4. Discussion

In order to accommodate the curvature and angles of vessels during delivery, it is important for stents to bend in order to provide optimal stent performance [19]. Longitudinal stent flexibility is an important parameter affecting stent delivery and how well the expanded stent conforms to the vessel wall. Flexible stents decrease the risk of vascular trauma and poorer flexibility increases the chance that postoperative thrombus will form [20]. It can be said that good stent flexibility helps clinicians treat vascular stenosis. At the same time, it also serves as a predictor for major adverse cardiac events [21]. Therefore, it is important to improve stent flexibility compared to conventional stents in order to prevent restenosis. Szabadits et al. [22] found that the flexibility of stents depends on stent design more than the raw materials. Hence, we focused on the flexibility of BDSs from the perspective of stent design.

The experimental results show that there is a difference in the flexibility of the four stents, but this difference is not significant. This may be explained by considering the different stent geometries and their variations during bending. Computational analyses can further enhance our understanding of stent performance. Although the simulation results are slightly different from the experimental results in terms of the numerical value, the rankings are consistent. We could not control some systematic errors in the simulation and experiment, such as the loading position and the computer simulation cannot simulate experiments perfectly. Therefore, we believe that the small differences between the numerical values obtained from the experimental and simulated data are acceptable. In addition, the numerical simulation shows that the peak stress areas after the stent is bent are mainly concentrated around the links with the largest deformation rather than the strut. Since all the stents use the same links, their flexibility does not vary significantly. In addition to the different strut configurations, all of the other parameters for the four types of stents were the same. Therefore, we can infer that the flexibility of the BDSs is related to the geometries of the struts and links. However, the geometries of struts have less effect on the flexibility and the links have more effect on the flexibility. The parameter of the links plays a more important role in the flexibility of the stent [8].

Links can improve stent flexibility but also reduce coverage [23]. The links between the struts increase the space for each unit in the stent and reduce the coverage of the stent [6]. A pathologic study demonstrated that the most powerful histological predictor of stent thrombosis is coverage [24]. Therefore, it is important to find a balance between stent flexibility and coverage. In addition, some studies have found that the flexibility of stents is also related to the connection mode, thickness, width and length of the links [9].

Although the relationship between the flexibility of the stent and the strut configuration is not significant, we can still make some qualitative conclusions based on the results. The experimental data shows that T1 and T4, with circular arc and spline curves respectively, had the best flexibility. In addition, the linear stent T2 was second best and the hexagonal stent T3 was the worst. The flexibility ranking from the numerical simulation and experimental results were basically consistent. The strut in T4 was designed using non-uniform rational B-splines (NURBS); therefore, the ring section of T4 was uneven, which may explain why it was easier to deform. However, the uneven design will also reduce the strength of stent at narrower parts of the struts [25]. Although strut configuration has no significant effect on stent flexibility, it may have significant effect on other properties, such as the radial support force and rebound rate [17].

In the results, we also conducted a force analysis of the stent strut in the process of three-point bending. This showed that the moment *M* leading to the deformation of the strut was proportional to the area enclosed by the strut centerline *A*, as shown in Figure 7. Therefore, the larger the area the larger moment, the larger the deformation of the stent, and the greater the flexibility of the stent. In contrast, the rank of *A* for all four stents is consistent with the experimental and numerical results. Thus, it can be concluded that the greater the area enclosed by the strut centerline, the better its flexibility. We suggest that in future stent designs, when the other necessary conditions are met, the area should be increased as much as possible in order to improve flexibility.

## 5. Conclusions

Optimizing the mechanical properties of stents can improve their long-term efficacy. It has been demonstrated that the geometry of stents plays an important role in their behavior, especially in terms of flexibility. This study helps to improve our understanding of key design features related to flexibility, particularly in regard to the geometry of struts. These results can be utilized in future research into the optimization of stent design using PLLA materials. In conclusion, the bending method and experimental results can be used to study the flexibility of stents with different struts. In addition, this could provide a basis for future research to help designers develop new stent designs with high flexibility based on the analysis of the mechanical characteristics of existing stents.

### Limitations of This Work

The study has some limitations. The stents used all had the same links and only the struts were different, so differences in the flexibility were not obvious. Furthermore, there may be some differences from reality due to the simplified model used; however, in terms of qualitative analysis, the trend would be similar. Our next step will be to study the relationship between the configuration of different links and the flexibility of BDSs. Another limitation is that bending was only in one direction and the crimping and expansion procedures of the stent were not considered before the bending test. Furthermore, the radial strength of biodegradable stents is the key of the research and the precondition of the study on the flexibility. Although this paper does not involve the discussion of radial strength, the radial strength of four kinds of stents in this paper has been studied in our previous research [18]. We will consider these aspects in future study.
